# Dry Sliding Behavior of an Aluminum Alloy after Innovative Hard Anodizing Treatments

**DOI:** 10.3390/ma14123281

**Published:** 2021-06-14

**Authors:** Chiara Soffritti, Annalisa Fortini, Anna Nastruzzi, Ramona Sola, Mattia Merlin, Gian Luca Garagnani

**Affiliations:** 1 Department of Engineering, University of Ferrara, via Saragat 1, 44122 Ferrara, Italy; chiara.soffritti@unife.it (C.S.); anna.nastruzzi@edu.unife.it (A.N.); mattia.merlin@unife.it (M.M.); gian.luca.garagnani@unife.it (G.L.G.); 2 Department of Industrial Engineering, University of Bologna, viale Risorgimento 4, 40136 Bologna, Italy; ramona.sola@unibo.it

**Keywords:** hard anodizing treatment, silver ions, microstructure, mechanical properties, wear

## Abstract

This work evaluates the dry sliding behavior of anodic aluminum oxides (AAO) formed during one traditional hard anodizing treatment (HA) and two golden hard anodizing treatments (named G and GP, respectively) on a EN AW-6060 aluminum alloy. Three different thicknesses of AAO layers were selected: 25, 50, and 100 μm. Prior to wear tests, microstructure and mechanical properties were determined by scanning electron microscopy (VPSEM/EDS), X-ray diffractometry, diffuse reflectance infrared Fourier transform (DRIFT-FTIR) spectroscopy, roughness, microhardness, and scratch tests. Wear tests were carried out by a pin-on-disc tribometer using a steel disc as the counterpart material. The friction coefficient was provided by the equipment. Anodized pins were weighed before and after tests to assess the wear rate. Worn surfaces were analyzed by VPSEM/EDS and DRITF-FTIR. Based on the results, the GP-treated surfaces with a thickness of 50 μm exhibit the lowest friction coefficients and wear rates. In any case, a tribofilm is observed on the wear tracks. During sliding, its detachment leads to delamination of the underlying anodic aluminum oxides and to abrasion of the aluminum substrate. Finally, the best tribological performance of G- and GP-treated surfaces may be related to the existence of a thin Ag-rich film at the coating/aluminum substrate interfaces.

## 1. Introduction

Aluminum and its alloys have been widely used in many industries due to their low density, lightweight, high thermal and electrical conductivities, and specific strength. Nevertheless, the low hardness and poor wear resistance of these materials limit their application range to parts serving only in the absence of friction [1,2].

It is well known that surface modification enhances the tribological properties of metal alloys [3,4,5,6,7,8]. With respect to aluminum and its alloys, the use of processes such as conventional and hard anodizing [9], thermal spraying [10], and plasma electrolytic oxidation [11] allows overcoming issues concerning their insufficient friction properties.

The aluminum alloys 6xxx series are becoming increasingly important in the aerospace and automotive industries due to their suitable physical properties such as weldability and formability coupled with the low cost. Similarly, anodizing is usually employed as an engineering solution for improving the tribological behavior of these aluminum alloys [2]. Conventional and hard anodizing are electrochemical treatments that originated in the 1930s, both consisting of the conversion of aluminum into its oxide by a proper selection of the type of electrolytic bath, temperature, voltage, and current density [12]. Conventional anodizing usually produces aluminum oxides up to 25 μm in thickness, whereas hard anodizing generates thicknesses between 25 and 150 μm. During the processes, an inert cathode and the component to be treated (anode) are placed in an electrolytic bath typically made up of an aqueous solution of sulfuric acid. The cathode is connected to the negative terminal of a voltage source, whereas the anode is connected to the positive one. When the electrical current flows through the circuit, the water in the electrolytic bath breaks down, and oxygen is deposited at the anode. This oxygen combines with aluminum to form a porous layer of anodic aluminum oxide (AAO), which is mainly composed of amorphous Al_2_O_3_ [13]. By adjusting the anodizing conditions, almost any desired properties of the AAO layer can be obtained, from thick, hard, and outstanding corrosion-resistant oxides for engineering applications to the thin films used for decorative purposes [9,14,15,16,17,18,19,20,21].

In the literature, the wear resistance of anodized aluminum alloys has been investigated in dry sliding conditions and under applied loads ranging from 1 mN to 10 N. Kim et al. [22] studied the friction and wear behavior of nanostructured AAO layers with various pore sizes in contact with a 440C stainless steel ball and under applied loads from 1 mN to 1 N. The authors showed that regardless of the pore size, the friction coefficient decreased with increasing load due to the formation of thick and smooth tribolayers at the higher loads. Guezmil et al. [23] determined the mechanical and tribological properties of a Al 5754 aluminum alloy in contact with a 100Cr6 steel ball under different normal loads (1, 2, and 3 N), sliding speeds (100, 200, and 300 tr/min) and thicknesses of the AAO layer (20 and 60 μm) by a pin-on-disk tribometer. In contrast to the previous study, this one demonstrated that the friction coefficient increased with increasing load and sliding speed. The AAO layer with low thickness seemed also more effective in terms of friction. Malayoglu et al. [24] investigated the mechanical and tribological behavior of a hard anodized AA6082 aluminum alloy in comparison with those of the same alloy after coating by the Keronite plasma electrolytic oxidation process. The wear tests were conducted at 10 and 20 N applied loads by applying sliding speeds of 0.01, 0.02, 0.06, and 0.10 ms^−^^1^, and using silicon nitride balls as the counterpart material. The wear resistance of the anodic aluminum oxide against corundum balls was evaluated by Tsyntsaru et al. [25] through wear tests in ball-on-flat configuration and under normal loads ranging from 40 mN to 1 N. Based on the results, AAO exhibited a very high friction coefficient; moreover, wear caused the formation of a thick, compacted and non-uniformly distributed tribolayer over the wear track. This layer induced a high adhesion between the anodic aluminum oxide and the corundum counterpart. Guezmil et al. [26] tested the performance of 1050A and 5754H111 aluminum alloys undergoing different anodizing conditions under a normal load of 1 N and against a 100Cr6 steel ball. The authors found that wear was affected by many factors, including initial morphology, chemical composition, porosity, and internal stresses of the AAO layer. Hierarchical AAO layers with improved wear resistance were successfully fabricated by Vengatesh et al. [27], obtaining values of the friction coefficient lower than 0.2 when tested under an applied load of 10 N and against a SS 301 stainless steel. Normal loads of 6, 8, and 10 N were selected by Dejun et al. [28] for studying the tribological properties of the anodic aluminum oxide against a ceramic ball. The results showed a decrease in friction coefficient up to 13.93% with increasing load. Lu et al. [29] evaluated the friction and wear behavior of AA2024 aluminum alloy anodized in a novel electrolytic bath after wear tests under an applied load of 10 N. Based on the results, the surface properties of the AAO layers strongly affected the wear resistance of the samples examined. Recently, micro-textured and anodized Al-Si alloys were proved at a normal load of 20 N against a nodular cast iron [30], whereas 5 N and corundum balls were selected by Benea et al. [31] to investigate the wear resistance of AAO layers grown using different anodizing conditions. In the last case, the wear rates were always lower than 2 × 10^−^^3^ mm^3^/Nm.

The anodic aluminum oxide is also potentially interesting for tribological applications, since its porosity can be used as a reservoir or template for lubricants to form self-lubricating structures [32,33,34]. The wear resistance of AAO layers filled in with solid or liquid lubricants has been studied to some extent [35,36,37]. Some authors proved that the infiltration of MoS_2_ into the porosity of anodic aluminum oxide improved the friction and wear behavior of aluminum alloys [38,39,40]. In addition, porous AAO layers with incorporated mineral oil showed better wear characteristics in comparison with unfilled ones [41].

In this study, two types of golden hard anodizing treatment indicated as G and GP were performed on EN AW-6060 aluminum alloy. In comparison with traditional hard anodizing processes, these innovative treatments involve the sealing of the porosity of the anodic aluminum oxides with silver ions by immersion in hot deionized water at different temperatures. The AAO layers obtained by G treatment have shown improved self-lubricating, antistatic, and antibacterial properties, corrosion resistance, and thermal conductivity [42]. However, very limited research has been conducted on the tribological behavior of anodic aluminum oxides after G treatment under dry sliding conditions [43]. Therefore, this study aims to evaluate the dry sliding behavior of AAO layers formed during G and GP in comparison with the behavior of the same layers obtained by a traditional hard anodizing treatment (HA). The microstructure and mechanical properties of anodic aluminum oxide layers were first determined by optical microscopy, scanning electron microscopy equipped with energy-dispersive spectroscopy, X-ray diffractometry, roughness, Vickers microhardness, and scratch tests. After wear tests by a pin-on-disc tribometer, the main wear mechanisms were investigated by Fourier transform infrared spectroscopy and scanning electron microscopy.

## 2. Materials and Methods

### 2.1. Microstructure and Mechanical Properties

The spherically ended pins of 6 mm in diameter and 30 mm in length used in this investigation were made of EN AW-6060 aluminum alloy with the following chemical composition (wt %): 0.470% Si, 0.260% Fe, 0.050% Cu, 0.075% Mn, 0.440% Mg, 0.009% Cr, 0.004% Ni, 0.054% Zn, 0.011% Ti, 0.010% Ga, 0.008% V, 0.001% Pb, balance Al. The chemical composition of pins was determined by optical emission spectroscopy (OES) through a SPECTROLAB analyzer (SPECTRO Analytical Instruments GmbH, Kleve, Germany).

A commercial company performed on pin surfaces the examined anodizing treatments (HA, G, and GP). Before anodizing, the surfaces to be treated were degreased and chemically cleaned in an alkaline solution. Hard anodizing treatment was carried out in a sulfuric acid bath at a voltage between 15 and 100 V, current density from 1 to 5 A/dm^2^, process temperature between −10 and +10 °C, and with a coating rate from 0.5 to 1.5 μm/min. The same voltage, current density, and coating rate were also adopted for G and GP treatments, but with a process temperature between −5 and +5 °C and using sulfuric acid with the addition of silver compounds as electrolyte. The post-anodizing treatment (sealing) of the anodic aluminum oxides obtained by HA and G was conducted in hot deionized water at 100 °C, whereas that of AAO layers produced by GP was performed in the same bath at lower temperature. For each treatment, three different thicknesses of the anodic aluminum oxide layers were selected: 25, 50, and 100 μm. Accordingly, the samples were divided into three groups: (i) HA_25, HA_50, and HA_100; (ii) G_25, G_50, and G_100; (iii) GP_25, GP_50, and GP_100. A summary of the samples is reported in Table 1, together with information on the types of anodizing treatment, their designation, and thicknesses of AAO layers.

The phase compositions of anodic aluminum oxide layers were studied by X-ray diffractometry (XRD) and diffuse reflectance infrared Fourier transform (DRIFT-FTIR) spectroscopy. The XRD analyses were carried out using a Philips X’PERT PW3050 diffractometer (Philips, Amsterdam, Netherlands), with Cu K-α radiation (λ = 1.54 Å), a pattern acquisition from 10° to 125° (2θ mode, 0.02° step size, 2 s/step scanner velocity and 1.5 grid), a 40 kV voltage, and a 40 mA filament current. The DRIFT-FTIR investigations were conducted with a Thermo-Scientific Nicolet iS50 spectrometer (Thermo Fisher Scientific, Waltham, USA). The spectrometer was purged with dry, CO_2_-free air generated with a Balston 75-52 unit, and a deuterated triglycine sulfate (DTGS) detector was used to investigate the region from 4000 to 800 cm^−1^ with a resolution of 4 cm^−1^. After polishing, cross-sections of pins were observed by a Zeiss EVO MA 15 (Zeiss, Oberkochen, Germany) scanning electron microscope operating in variable pressure conditions and coupled with an Oxford X-Max 50 (Oxford Instruments, Abingdon-on-Thames, UK) microprobe for energy-dispersive spectroscopy (VPSEM/EDS). The operating parameters of the scanning electron microscope were 20 kV acceleration voltage in air atmosphere at a 50 Pa pressure. Aluminum alloy before anodizing treatments and all anodized surfaces were first cleaned in an ultrasonic bath; then, the roughness parameters (R_a_ and R_z_) were evaluated by a Talysurf CCI-Lite non-contact 3D profilometer (Taylor-Hobson, Leicester, UK). The Vickers microhardness was measured in triplicate before and after anodizing treatments on the cross-sections of pins under 10 g_f_ load and 15 s loading time (HV0.01) by a Future-Tech FM-110 Vickers microindenter (Future-Tech Corp., Kawasaki, Japan). The scratch tests were conducted on the anodized surfaces by a MICRO COMBI Scratch Tester (CSM Instrument, Needham, MA, USA), with a 200 μm Rockwell C diamond indenter, a linearly increased load from 20 mN to 30 N, a scratching speed of 5 mm/min, and a scratch length of 5 mm. A mean of five scratches was performed for each anodizing treatment and thickness of the AAO layers. The graphs of normal force, acoustic emission, and penetration depth vs. scratch distance, provided by the instrument, were analyzed to evaluate the critical loads L_c1_ and L_c2_. After the tests, the scratches were also examined under a Leica MEF4 M (Leica, Wetzlar, Germany) optical microscope to identify the position at which the AAO layers had peeled off.

### 2.2. Wear Tests

Wear tests were carried out by a TR-20LE tribometer (Ducom Instruments, Bengaluru, India) in pin-on-disc configuration (ASTM G99-17), using a 100Cr6 steel disc of 165 mm in diameter as a counterpart material. The tests were conducted under an applied load of 10 N, at a sliding velocity of 0.1 m/s, at room temperature, and in dry conditions. A sliding distance of 250 m was selected. For each anodizing treatment and thickness of the AAO layers, five wear tests were performed. The friction coefficient was provided by the equipment. To assess the wear rate (WR), anodized pins were weighed before and after tests on an AE240 analytical balance (Mettler Toledo, Columbus, OH, USA), with a resolution of 0.01 mg and a reproducibility value of ±0.05 mg. The balance was positioned in a controlled temperature room, with a controlled air temperature of 22 ± 1 °C. The weighing process was repeated five times through an independent weighing measure process. Then, weight loss was used to calculate the volume loss by dividing it by the material density. To identify the main wear mechanisms, the worn surfaces on pins were examined by VPSEM/EDS with the Zeiss EVO MA 15 scanning electron microscope (Zeiss) coupled with the Oxford X-Max 50 microprobe (Oxford Instruments). Finally, wear debris collected from the pins were investigated by DRIFT-FTIR with the Thermo-Scientific Nicolet iS50 spectrometer (Thermo Fisher Scientific).

## 3. Results

### 3.1. Microstructure and Mechanical Properties

**Figure 1:**
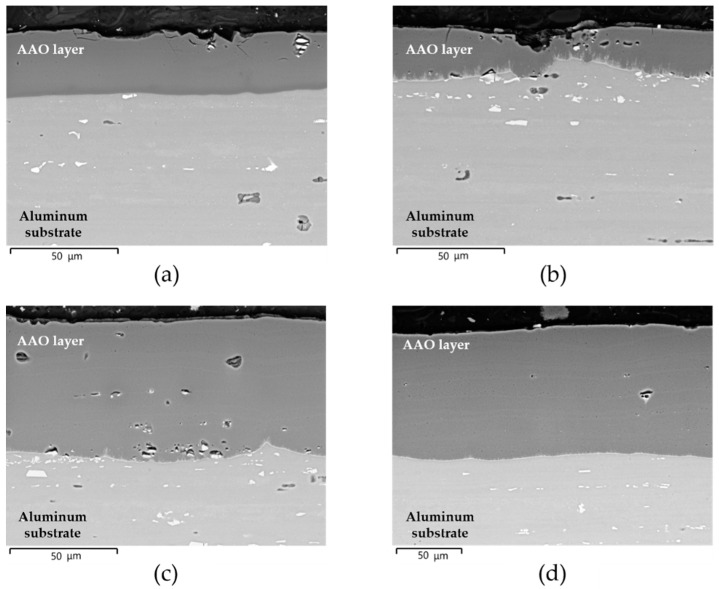
VPSEM micrographs in back-scattered electron mode of microstructure observed on the cross-section of pins before wear tests: (**a**) HA_25; (**b**) G_25; (**c**) G_50; (**d**) GP_100.

The VPSEM micrographs in the back-scattered electron mode of microstructures observed on the cross-section of pins before wear tests are shown in Figure 1a–d.

**Figure 2:**
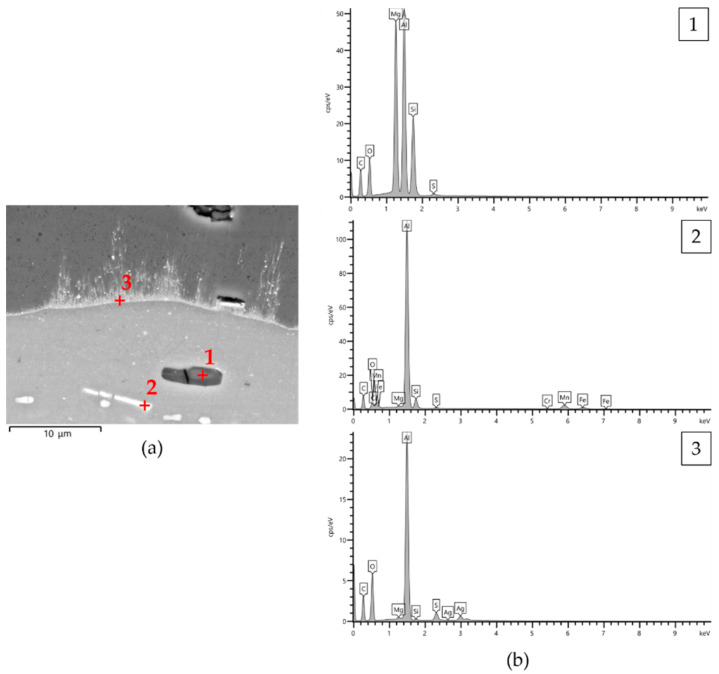
(**a**) VPSEM micrograph in back-scattered electron mode of microstructure observed on the cross-section of GP_25 pins before wear tests and (**b**) semi-quantitative EDS analyses (wt %) of different areas in the same microstructure.

All anodic aluminum oxides exhibited well-bonded coating/aluminum substrate interfaces. Irrespective of the type of anodizing treatment, the surfaces of pins with a thickness of AAO layers of 25 μm were partially irregular with some microcracks propagating toward the interior of the anodic aluminum oxide (Figure 1a,b). For other thicknesses, the microcracks were still visible, but the surfaces of pins were less irregular (Figure 1c,d). All AAO layers showed many micro-porosities above intermetallic particles originating from the aluminum substrate, which were included within the coatings during anodizing treatments. These particles appear as dark and light contrast areas of size less than 15 μm in Figure 2. The EDS analyses revealed the presence of aluminum, magnesium, silicon, and oxygen in the dark contrast areas, whereas aluminum, silicon, magnesium, iron, manganese, and chromium were detected in the light contrast areas (spectra 1 and 2 in Figure 2, respectively). At the coating/aluminum substrate interfaces of pins with G and GP treatments, very fine-scale dendrites protruding inside the anodic aluminum oxides were also observed. It should be mentioned that their extent decreased with the increasing thickness of the AAO layers. Comparing the semi-quantitative EDS analyses of the elements in the dendrites and in different regions of pin surfaces, the dendrites contained silver (spectrum 3 in Figure 2).

**Figure 3:**
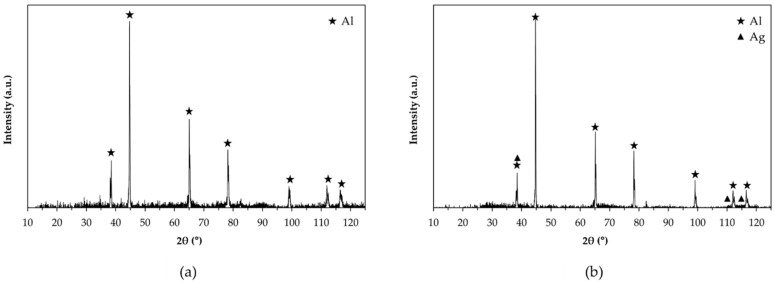
X-ray diffraction patterns recorded on the surface of pins before wear tests: (**a**) HA_50; (**b**) GP_100.

**Figure 4:**
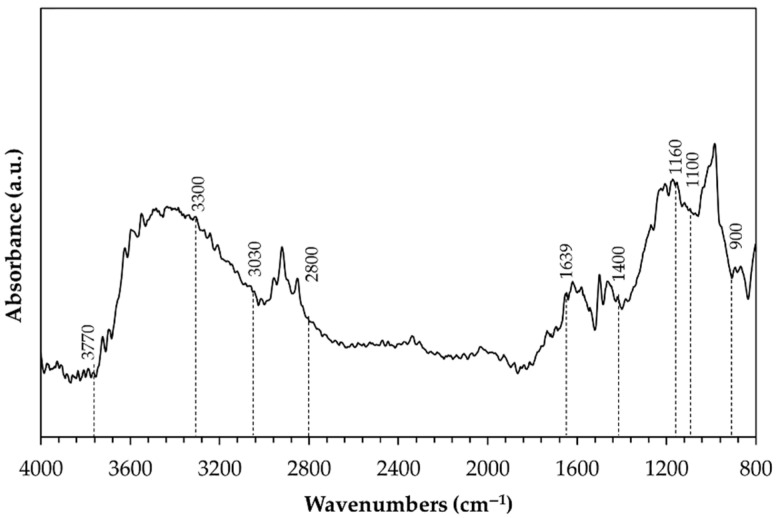
Representative DRIFT-FTIR spectrum of all pin surfaces before wear tests.

The X-ray diffraction patterns recorded on the surface of pins before wear tests are shown in Figure 3. No crystalline phases were identified due to the amorphous structure of anodic aluminum oxide. Only aluminum peaks deriving from the substrate appeared in the diffractograms of the surfaces with HA treatment (Figure 3a), while the same peaks plus that of silver were detected for G- and GP-treated surfaces (Figure 3b). A representative DRIFT-FTIR spectrum of all pin surfaces before wear tests is reported in Figure 4. A broad band in the range of 3030–3770 cm^−1^ was detected, corresponding to OH stretching of hydroxyl groups in the amorphous structure of the AAO layers. The shoulder around 1639 cm^−1^ was considered the bending mode of absorbed water. In the OH bending region, the spectrum was characterized by one vibration at 1160 cm^−1^ and by an additional band between 1100 and 900 cm^−1^, the latter assigned to bending vibrations of Al-OH. Traces of organic compounds were also detected (peaks at 2800–3000 cm^−1^ and around 1480 and 1380 cm^−1^), which were probably due to contamination.

The mean values ± standard deviations of the roughness parameters (R_a_ and R_z_) and Vickers microhardness (HV0.01) for all anodizing treatments and thicknesses of AAO layers are reported in Table 2. The mean values of R_a_ and R_z_ of aluminum alloy before anodizing treatments were 0.16 ± 0.01 μm and 1.5 ± 0.38 μm, respectively. After anodizing treatments, slight differences were found in the mean values of R_a_ (between 0.17 and 0.23 μm) and R_z_ (ranging from 1.65 to 2.16 μm) of all surfaces examined. The mean value of the Vickers microhardness of aluminum alloy before anodizing treatments was 115 ± 3 HV0.01. After anodizing treatments, irrespective of the thickness, the anodic aluminum oxide obtained with the traditional hard anodizing treatment showed the highest Vickers microhardness (between 438 and 458 HV0.01), whereas that of the AAO layers generated by the GP treatment was the lowest (ranging from 356 to 412 HV0.01). For all anodizing treatments, the Vickers microhardness values of the anodic aluminum oxide with a thickness of 50 μm were higher than those of the AAO layers that were 25 and 100 μm thick.

**Figure 5:**
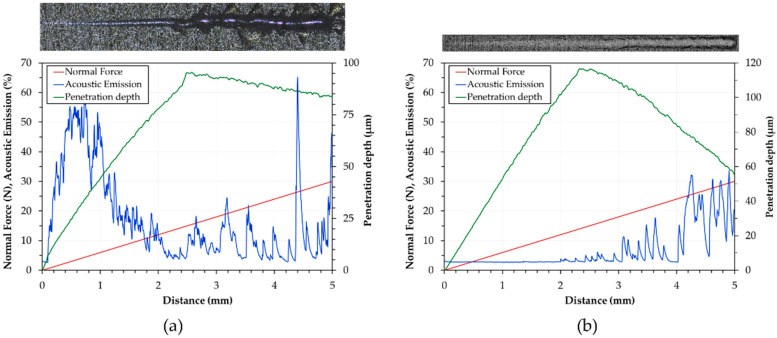
Optical micrographs of scratches on pin surfaces with anodic aluminum oxides of (**a**) 25 and (**b**) 50 μm, and the corresponding graphs of normal force, acoustic emission, and penetration depth vs. scratch distance.

**Figure 6:**
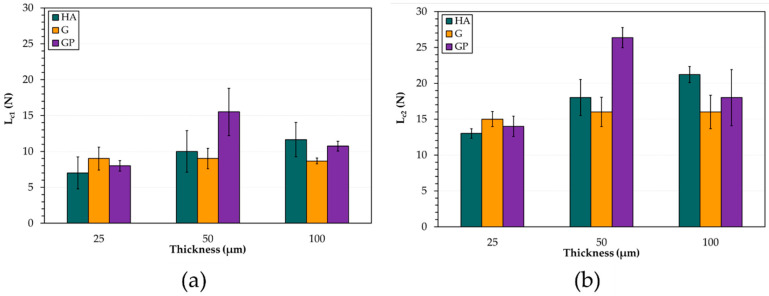
Mean values ± standard deviations of the critical loads (**a**) L_c1_ and (**b**) L_c2_ for all anodizing treatments and thicknesses of anodic aluminum oxides.

The optical micrographs of scratches on pin surfaces with anodic aluminum oxides of 25 and 50 μm, and the corresponding graphs of normal force, acoustic emission, and penetration depth vs. scratch distance, are reported in Figure 5. For all AAO layers with a thickness of 25 μm, the acoustic emission variations did not show any identifiable point at which the failure of the anodic aluminum oxides could occur. The penetration depths increased with increasing scratch distance and reached a maximum value of about 98 μm; then, they progressively decreased until the end of the tests (Figure 5a). Conformal cracks were detected within the scratches, before the occurrence of the adhesive failure of the AAO layers (optical micrograph in Figure 5a). For all anodic aluminum oxides with thicknesses higher than 25 μm, the acoustic emission variations showed two distinct regions indicating different levels of damage of the AAO layers (Figure 5b). Concerning the penetration depths, the curves were similar to those of the coatings 25 μm thick but with maximum values registered of about 116 and 95 μm for the thickness of 50 and 100 μm, respectively. The mean values ± standard deviations of the critical loads (L_c1_ and L_c2_) for all anodizing treatments and thicknesses of anodic aluminum oxides are reported in Figure 6. Irrespective of the type of anodizing treatment, AAO layers with a thickness of 25 μm exhibited mean values of L_c1_ and L_c2_ lower than those of the coatings with anodic aluminum oxides of 50 and 100 μm. For these thicknesses, the highest L_c1_ and L_c2_ were 16 and 26 N, which were both registered for the GP treatment.

### 3.2. Wear Tests

**Figure 7:**
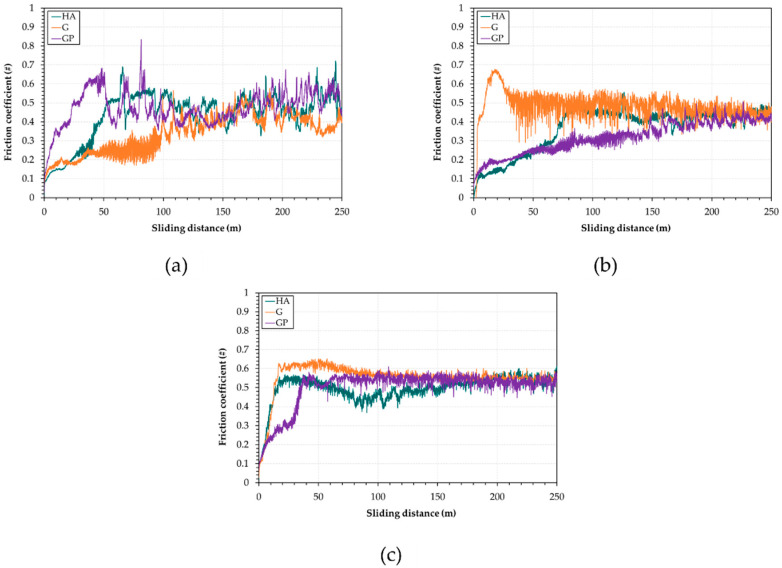
Friction coefficient variations calculated during the tests for the different anodizing treatments and for thicknesses of AAO layers of (**a**) 25 μm; (**b**) 50 μm; (**c**) 100 μm.

The friction coefficient variations calculated during the tests for the different anodizing treatments and thicknesses of AAO layers are depicted in Figure 7.

For anodic aluminum oxides with a thickness of 25 μm (Figure 7a), the friction coefficients gradually increased and achieved the quasi-steady state (μ = 0.40–0.50) at sliding distances between 60 and 100 m. Once this state was attained, the friction coefficients also displayed large oscillations around the average values. For a thickness of AAO layers of 50 μm (Figure 7b), the G-treated surfaces underwent severe running-in wear until 40 m, after which μ decreased to about 0.47. Then, this value remained the same until the end of the tests. Comparing the friction coefficients curves of G- and HA- plus GP-treated surfaces, the last ones exhibited less severe running-in wear. The anodic aluminum oxides obtained after HA treatment reached the quasi-steady state (μ ≅ 0.43) after 80 m, whereas μ values of the AAO layers generated by GP treatment progressively increased during tests. For a thickness of AAO layers of 100 μm (Figure 7c), irrespective of the type of anodizing treatment, the friction coefficients rose up to 0.56–0.61 during the first few minutes. Then, the μ values slightly decreased up to 0.54, from a sliding distance of about 150 m onward.

**Figure 8:**
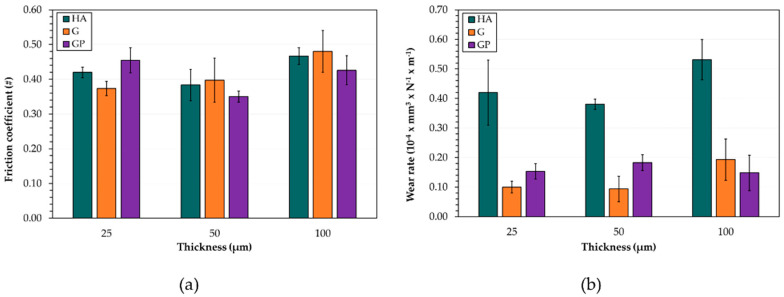
Mean values ± standard deviations of (**a**) friction coefficient and (**b**) wear rate for all anodizing treatments and thicknesses of anodic aluminum oxides.

The mean values ± standard deviations of (a) friction coefficient and (b) wear rate for all anodizing treatments and thicknesses of anodic aluminum oxides are reported in Figure 8. Regardless of the type of anodizing treatment, AAO layers with a thickness of 50 μm showed the lowest friction coefficient (μ = 0.35–0.40) (Figure 8a) and wear rate (WR = 9.38 × 10^−6^–3.80 × 10^−5^ mm^3^/Nm). When all thicknesses were considered, μ and WR increased in the following order: 50 < 25 < 100 μm. Considering the anodic aluminum oxides with a thickness of 25 μm, the lowest friction coefficient was that of the G-treated surfaces (μ = 0.37), whereas for thicknesses of 50 and 100 μm, the best μ performances were achieved after GP treatment (μ = 0.35 and μ = 0.43, respectively) (Figure 8a). Finally, the wear rates of AAO layers produced by G and GP treatments were similar and, in any case, they were lower than those of anodic aluminum oxides generated by HA treatment (Figure 8b).

**Figure 9:**
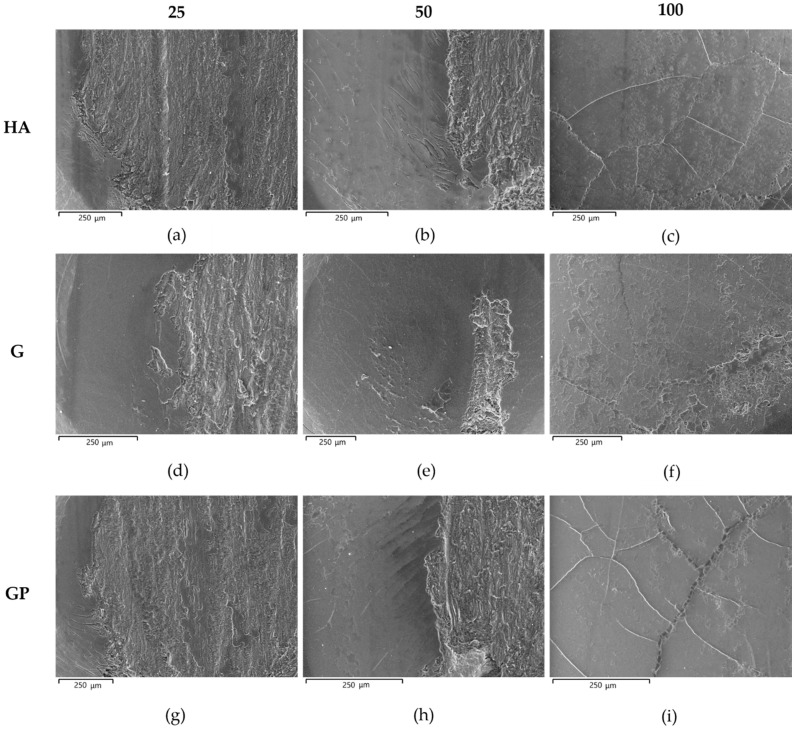
VPSEM images of the worn surface of pins after wear tests: (**a**–**c**) HA-, (**d**–**f**) G-, and (**g**–**i**) GP-treated surfaces with all thicknesses examined.

**Figure 10:**
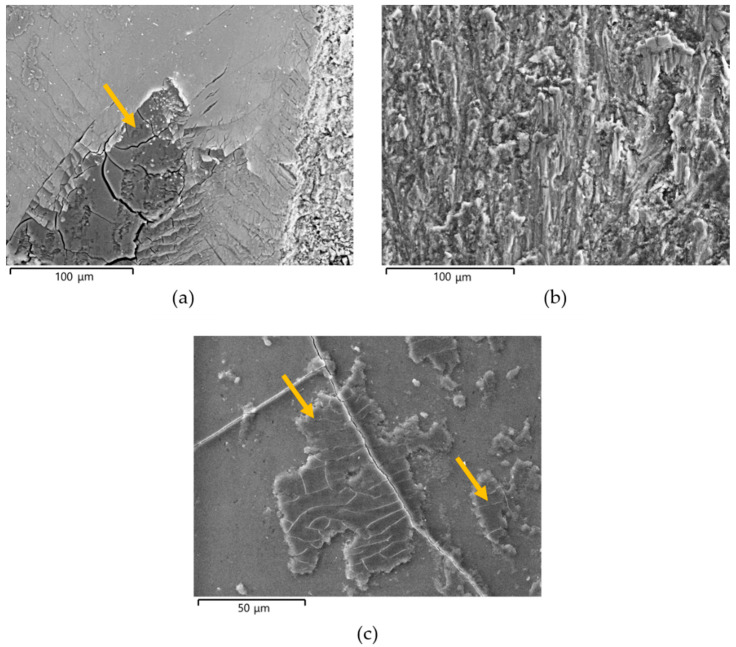
VPSEM images of the worn surfaces of pins after wear tests: (**a**) details of the original surface with metallic film deposition on G_50; (**b**) details of abrasion of the aluminum substrate in GP_50; (**c**) details of metal transfer and microcracks on G_100. In (**a**,**c**), the orange arrows indicate the tribofilm on the wear tracks, consisting of well-adherent, compacted, and plate-shaped wear particles.

**Figure 11:**
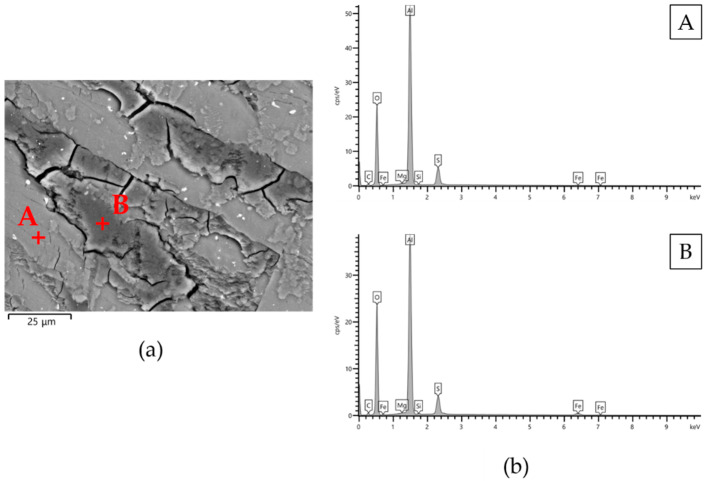
(**a**) VPSEM backscattered electron image of the worn surface of pins underwent G_50 anodizing treatment and (**b**) semi-quantitative EDS analyses of (**A**) tribofilm and (**B**) the first region of the wear tracks.

**Figure 12:**
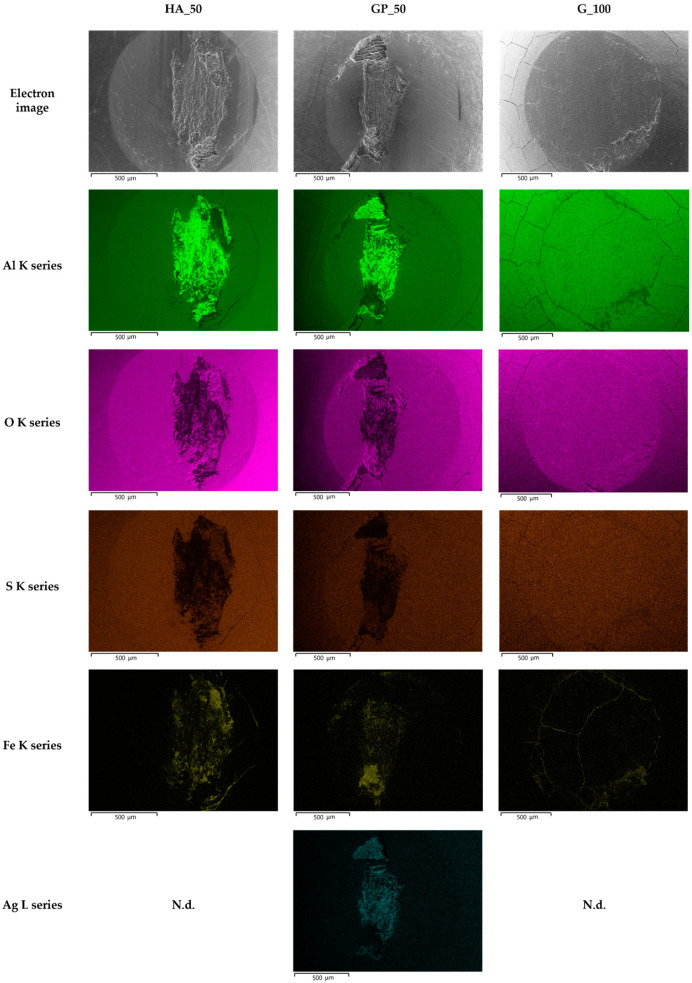
VPSEM/EDS backscattered electron images and maps of elemental distribution on the worn surfaces of pins HA_50, GP_50, and G_100. N.d. means not detected.

The VPSEM images of the worn surface of pins after wear tests are shown in Figure 9. Irrespective of the type of anodizing treatment, the worn surfaces of the coatings with thicknesses of 25 and 50 μm could be divided in two regions (Figure 9a,b,d,e,g,h). The first one was the original surface with metallic film deposition consisting of well-adherent, compacted and plate-shaped wear particles (details in Figure 10a). In addition, many microcracks formed during sliding motion were visible on the tribofilm and anodic aluminum oxides; however, AAO layers were partially cracked before the wear tests. Comparing the elements detected in the tribofilm and in the first region of the wear tracks, the former was richer in iron; in both cases, the EDS analyses also revealed the presence of aluminum, oxygen, sulfur, silicon, and magnesium (Figure 11). In the second region, the tribofilm was completely detached from the anodized surfaces. This detachment led to delamination of the underlying anodic aluminum oxides and to the subsequent abrasion of the aluminum substrate in the latter stages of wear tests (details in Figure 10b). The coatings with a thickness of 25 μm showed a wider second region than those with a thickness of 50 μm. For the AAO layers generated by G and GP treatments, the VPSEM/EDS element mapping allowed identifying enrichments of aluminum, iron, and silver on the abraded areas of the wear tracks; on the contrary, no traces of this latter element were found on the same regions of the HA-treated surfaces (Figure 12). Concerning the worn surfaces of the coatings with a thickness of 100 μm, only the first region was visible (Figure 9c,f,i), with the metal transfer preferentially located close to the microcracks on anodic aluminum oxides (details in Figure 10c). Accordingly, only elements deriving from anodizing treatments (e.g., aluminum, oxygen, and sulfur) plus iron were detected by VPSEM/EDS on the wear tracks (Figure 12).

**Figure 13:**
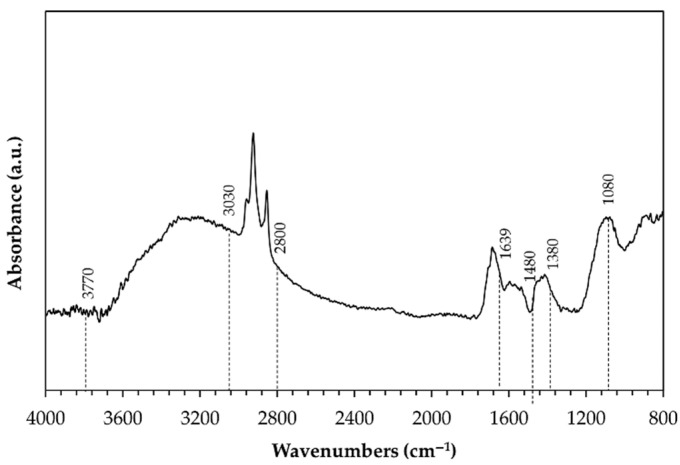
Representative DRIFT-FTIR spectrum of the wear particles collected after wear tests.

A representative DRIFT-FTIR spectrum of the wear particles collected after wear tests is reported in Figure 13. Comparing this spectrum with that in Figure 4, the broad band in the range of 3030–3770 cm^−1^, the shoulder at 1639 cm^−1^, and the peaks at 2800–3000 cm^−1^ and around 1480 and 1380 cm^−1^ were still visible. On the contrary, in the OH bending region, only one vibration appeared at 1080 cm^−1^.

## 4. Discussion

Wear tests were performed for investigating the dry sliding behavior of anodic aluminum oxides formed during traditional and innovative hard anodizing treatments on a EN AW-6060 aluminum alloy. The extensive metallographic investigation of pins before wear tests, which underwent HA, G, and GP treatments, shows the typical microstructure of anodic aluminum oxides [24,43]. All AAO layers are well-bonded to the underlying aluminum substrate. Their surfaces appeared more or less irregular, with microcracks propagating through the thickness of the coatings and many oxides inclusions (probably Al_2_O_3_ with the addition of MgO and SiO_2_) plus intermetallic particles (richer in silicon and containing iron, manganese, and chromium), which were incorporated during anodizing treatments. It is known that oxides inclusions may enter the melt at any stage of the foundry process when the molten metal is exposed to air or water vapor [44]. Concerning the intermetallic particles, their chemical composition is comparable to that previously reported for the aluminum alloys 6xxx series [1,2]. Studies on the formation and growth of AAO layers on Al-Si alloys also proved that the microporosities visible by VPSEM/EDS above Si-rich particles, with sizes and shapes that resemble particle morphologies, derive from localized stresses induced by the volume expansion around them [45,46]. Finally, very fine-scale and Ag-rich dendrites protruding inside the anodic aluminum oxides appear at the coating/aluminum substrate interfaces of pins, which underwent golden hard anodizing treatments.

Evaluation of the phase compositions of anodic aluminum oxides before wear tests highlights their amorphous structures and, for G- and GP-treated surfaces, confirms the presence of silver due to the post-anodizing treatment. In addition, further investigations by DRIFT-FTIR suggest that in all cases, AAO layers include some boehmite (aluminum oxyhydroxide, γ-AlO(OH)), since all spectra show the characteristic peaks of this hydrate phase [47,48,49,50,51,52], together with those that may be related to contamination. Boehmite is frequently encountered in anodic aluminum oxides as a hydration product of the same oxides and of the barrier layers, and it fills the pore during sealing in hot deionized water [53,54].

Based on the results of our mechanical characterization, the AAO layers generated by HA treatment are the hardest, while the anodic aluminum produced by GP treatment is the softest. Moreover, irrespective of the type of anodizing treatments, the anodic aluminum oxides with a thickness of 50 μm display mean values of HV0.01 higher than those of the coatings 25 and 100 μm thick. It should be pointed out that the Vickers microhardness of G-treated surfaces is slightly higher than that reported in Ref. [43]. The scratch tests demonstrate that wedge spallation is the main failure mode of the coatings with a thickness of 25 μm. This mode results in total delamination (adhesive failure) due to compressive stresses ahead of the indenter and through the thickness of the coatings; it should be mentioned that conformal cracking often precedes failure by wedge spallation, as confirmed by our optical microscope observations [55]. The adhesive failure probably follows the failure and/or the release of the elastic energy at the coating/aluminum substrate interface, as suggested by the wide initial variations seen in Figure 5a [55]. Finally, the variation of penetration depth vs. scratch distance (especially the decrease in the last stages of the scratch tests) suggests the occurrence of a pile-up phenomenon that is an accumulation of displaced plastic material around the last position of contact between the advancing indenter and the anodized surface [56]. For all anodic aluminum oxides with thicknesses of 50 and 100 μm, conformal cracking appears, and the anodic aluminum oxides remain adherent to the aluminum substrate. The conformal cracking mode involves cracking within the scratch only, along semicircular trajectories parallel to the leading edge of the indenter [57,58]. Previous studies indicate that possible reasons for this mode of failure may be the tensile stresses developed in the AAO layers behind the trailing edge of the indenter or the anodic aluminum oxides trying to conform to the shape of the scratch grooves [24]. Concerning critical loads, the lowest values of L_c1_ and L_c2_ for all coatings 25 μm thick are an index of their poor adhesion strength in comparison with that of the AAO layers 50 and 100 μm thick. Among these, the highest L_c1_ and L_c2_ are both registered for the GP-treated surfaces. All values of the critical loads are lower than those recorded for traditional and innovative treatments performed on AA6082 alloy [24,59].

For most anodizing treatments and thicknesses of anodic aluminum oxides, the friction coefficient variations with sliding distance display at least two different regimes, which are related to changes in wear morphology and degree of oxidation: the first one involves an increase in μ and the second is an achievement of a quasi-steady state. The results shown in Figure 7 are similar to those previously reported in [23,26,59]. According to the authors, the raising in friction coefficient during the first stage of wear tests can be attributed to the transition in contact conditions from the couple anodic aluminum oxide vs. steel to iron oxide vs. iron oxide, as further confirmed by our VPSEM/EDS observations. Irrespective of the type of anodizing treatment, coatings with a thickness of 50 μm show the lowest friction coefficients and wear rates. Some research established that this type of AAO layer is more effective in terms of friction, wear, and scratch resistance in dry sliding conditions [23,60]. Among the anodized surfaces 50 μm thick, the best tribological performance of the GP-treated ones may be associated with the highest values of L_c1_ and L_c2_. During sliding motion, the anodic aluminum oxides experience shear stresses, which may exceed the yield strength of the material [24]. For the GP treatment, AAO layers are able to comply with the deformation of the aluminum substrate induced by the elastic stresses and/or plastic strains at the near-surface regions of the coating. It should be highlighted that all values of μ and WR are comparable to those published in [43].

Scanning electron microscope observations of the wear tracks on the coatings with thicknesses of 25 and 50 μm support the formation of a smooth tribofilm consisting of well-adherent, compacted, and plate-shaped iron oxides, as identified by VPSEM/EDS. This metallic film deposition is produced by the combined effects of the tribochemical reaction that includes metal transfer from the steel disc over AAO layers and severe plastic deformation (mostly compression) of wear particles [22,24,26]. Previous research suggests that the strong adhesion of the tribofilm is provided by the asperities on the surface of the anodic aluminum oxides, owing to mechanical interlocking phenomena [24,43]. Many microcracks are also detected in the tribofilm and AAO layers, as a result of nucleation of new cracks and propagation of pre-existing ones, which was already observed before anodizing treatments by VPSEM/EDS. These microcracks are all due to shear stresses arisen during sliding. It is well-known that once the tribofilm is formed, it protects the underlying coatings until it reaches a critical thickness, as confirmed in our study by the achievement of a quasi-steady state in μ variations with the sliding distance [22,23,24]. When the critical thickness is attained, the metallic film deposition becomes unstable, fractures under stress cycling, and completely detaches from the anodized surfaces; the underlying coatings accordingly delaminate. This delamination, which affects more or less extended areas of the wear tracks, leads to abrasion of the aluminum substrate in the latter stages of the wear tests. No abraded regions are visible on the worn surfaces of the coatings with a thickness of 100 μm, but only the metal transfer mostly located close to the microcracks in anodic aluminum oxides. The EDS examinations of delaminated and abraded areas of G-and GP-treated surfaces (Figure 12) detect the presence of silver derived from the post-anodizing treatment with silver ions. The presence of this element at the coating/aluminum substrate interfaces is considered by the authors to be the main reason of the lower mean wear rates and possibly of the tendency toward lower mean friction coefficients of AAO layers generated by G and GP treatments, in comparison with those of anodic aluminum oxides produced by HA. Previous studies confirm that silver can act as a solid lubricant that is able to reduce friction and wear in a wide range of temperatures (from room temperature to about 500 °C) [61,62]. Indeed, silver has a tendency to plastically flow under high friction and pressure, forming a continuous lubrication film on the wear tracks. This condition is supported by our VPSEM/EDS analyses. Finally, the DRIFT-FTIR analysis of the wear debris collected from the pins after wear tests confirms that boehmite remains unchanged during sliding motion in all experimental conditions.

## 5. Conclusions

This work evaluates the dry sliding behavior of anodic aluminum oxides formed during innovative and traditional hard anodizing treatments on a EN AW-6060 aluminum alloy. The main results can be summarized as follows:

- All AAO layers show the typical microstructure of anodic aluminum oxides with very fine-scale and Ag-rich dendrites that appear only at the coating/aluminum substrate interfaces of G- and GP-treated surfaces. In agreement with the phase compositions analysis, all AAO layers exhibit amorphous structures with some boehmite deriving from the sealing in hot deionized water;
- The HA treatment generates the hardest anodic aluminum oxides, while GP produces the softest. The examination of scratches reveals two main failure modes: wedge spallation for the coatings 25 μm thick and conformal cracking for those 50 and 100 μm thick. Finally, all anodic aluminum oxides with a thickness of 25 μm display the lowest adhesion strength, whereas the GP-treated surfaces show the highest one;
- The friction coefficient variations with sliding distance are influenced by changes in wear morphology and the extent of oxidation. The GP-treated surfaces with a thickness of 50 μm exhibit the lowest friction coefficients and wear rates. Their improved wear resistance may be related to the increased adhesion strength compared to those of the other anodized surfaces;
- In any case, a well-adherent tribofilm consisting of iron oxide is observed on the wear tracks as a result of tribochemical reaction and metal transfer between the mating materials. During sliding, its detachment leads to delamination of the underlying anodic aluminum oxides and to the subsequent abrasion of the aluminum substrate. Despite the presence of abraded surfaces, the best tribological performance of G- and GP-treated surfaces may be related to the existence of a thin Ag-rich film at the coating/aluminum substrate interfaces.

## Figures and Tables

**Table 1:** Summary of the samples, together with information on the types of anodizing treatment, their designation, and thicknesses of AAO layers.

Types of Anodizing Treatment	Designation	Thicknesses of AAO Layers (μm)	Samples
Hard anodizing treatment	HA	25	HA_25
		50	HA_50
		100	HA_100
Golden hard anodizing treatment	G	25	G_25
		50	G_50
		100	G_100
Golden hard anodizing treatment_plus	GP	25	GP_25
		50	GP_50
		100	GP_100

**Table 2:** Mean values ± standard deviations of the roughness parameters (R_a_ and R_z_) and Vickers microhardness (HV0.01) for all anodizing treatments (HA, G, and GP) and thicknesses of AAO layers (25, 50, and 100 μm).

	25			50			100		
	R_a_ (μm)	R_z_ (μm)	HV0.01	R_a_ (μm)	R_z_ (μm)	HV0.01	R_a_ (μm)	R_z_ (μm)	HV0.01
HA	0.18 ± 0.02	2.09 ± 0.40	450 ± 20	0.22 ± 0.02	1.98 ± 0.33	458 ± 10	0.17 ± 0.01	1.81 ± 0.27	438 ± 11
G	0.19 ± 0.01	2.13 ± 0.37	427 ± 18	0.19 ± 0.05	2.06 ± 0.53	435 ± 12	0.17 ± 0.02	1.65 ± 0.13	398 ± 15
GP	0.23 ± 0.02	2.16 ± 0.35	374 ± 31	0.21 ± 0.05	2.14 ± 0.53	412 ± 17	0.18 ± 0.05	2.09 ± 0.48	356 ± 30

## Data Availability

Not applicable.

## References

[1] King F. (1987). Aluminium and Its Alloys.

[2] Anderson K., Weritz J., Gilbert Kaufman J. (2018). ASM Handbook, Volume 2A: Aluminum Science and Technology.

[3] Soffritti C., Fortini A., Merlin M., Garagnani G.L., Vazquez Aguilar R. (2019). Wear behaviour of a plasma-sprayed Al_2_O_3_-13%TiO_2_ ceramic coating/steel couple under high constant and variable loads. Metall. Ital..

[4] Soffritti C., Merlin M., Vazquez R., Fortini A., Garagnani G.L. (2018). Failure analysis of worn valve train components of a four-cylinder diesel engine. Eng. Fail. Anal..

[5] Soffritti C., Merlin M., Vazquez R., Garagnani G.L. (2018). Tribological behavior of a Cr_2_O_3_ ceramic coating/steel couple under dry sliding and heavy loading conditions. J. Mater. Eng. Perform..

[6] Merlin M., Soffritti C., Vazquez R. (2013). Effect of relative humidity and applied loads on the tribological behaviour of a steel/Cr_2_O_3_-ceramic coupling. Wear.

[7] Merlin M., Soffritti C., Vazquez R., Garagnani G.L. (2011). Friction and wear behaviour of APS and HVOF advanced ceramic coatings. Metall. Ital..

[8] Merlin M., Soffritti C., Vazquez R., Garagnani G.L. Effect of varying load on the tribological behaviour of a steel/ Al_2_O_3_-TiO_2_ ceramic coating. Proceedings of the 5th World Tribology Congress, WTC 2013.

[9] Bensalah W., Elleuch K., Feki M., DePetris-Wery M., Ayedi H.F. (2009). Comparative study of mechanical and tribological properties of alumina coatings formed on aluminium in various conditions. Mater. Des..

[10] Bolelli G., Lusvarghi L., Barletta M. (2009). HVOF-sprayed WC–CoCr coatings on Al alloy: Effect of the coating thickness on the tribological properties. Wear.

[11] Sabatini G., Ceschini L., Martini C., Williams J.A., Hutchings I.M. (2010). Improving sliding and abrasive wear behaviour of cast A356 and wrought AA7075 aluminium alloys by plasma electrolytic oxidation. Mater. Des..

[12] Young L. (1961). Anodic Oxide Films.

[13] Thompson G. (1997). Porous anodic alumina: Fabrication, characterization and applications. Thin Solid Films.

[14] Mezlini S., Elleuch K., Kapsa P. (2006). The effect of sulphuric anodisation of aluminium alloys on contact problems. Surf. Coat. Technol..

[15] López V., Otero E., Bautista A., González J. (2000). Sealing of anodic films obtained in oxalic acid baths. Surf. Coat. Technol..

[16] Jagminas A., Bigelien D., Mikulskas I., Tomašiūnas R. (2001). Growth peculiarities of aluminum anodic oxide at high voltages in diluted phosphoric acid. J. Cryst. Growth.

[17] Lunder O., Walmsley J.C., Mack P., Nisancioglu K. (2005). Formation and characterisation of a chromate conversion coating on AA6060 aluminium. Corros. Sci..

[18] Shih H.-H., Tzou S.-L. (2000). Study of anodic oxidation of aluminum in mixed acid using a pulsed current. Surf. Coat. Technol..

[19] Sulka G.D., Parkoła K.G. (2006). Anodising potential influence on well-ordered nanostructures formed by anodisation of aluminium in sulphuric acid. Thin Solid Films.

[20] Moutarlier V., Gigandet M., Pagetti J., Normand B. (2004). Influence of oxalic acid addition to chromic acid on the anodising of Al 2024 alloy. Surf. Coat. Technol..

[21] Bensalah W., Elleuch K., Feki M., Wery M., Ayedi H.F. (2007). Optimization of anodic layer properties on aluminium in mixed oxalic/sulphuric acid bath using statistical experimental methods. Surf. Coat. Technol..

[22] Kim H., Kim D., Lee W., Cho S.J., Hahn J.-H., Ahn H.-S. (2010). Tribological properties of nanoporous anodic aluminum oxide film. Surf. Coat. Technol..

[23] Guezmil M., Bensalah W., Khalladi A., Elleuch K., De-Petris Wery M., Ayedi H.F. (2014). Effect of test parameters on the friction behaviour of anodized aluminium alloy. Int. Sch. Res. Not..

[24] Malayoglu U., Tekin K.C., Malayoglu U., Shrestha S. (2011). An investigation into the mechanical and tribological properties of plasma electrolytic oxidation and hard-anodized coatings on 6082 aluminum alloy. Mater. Sci. Eng. A.

[25] Tsyntsaru N., Kavas B., Sort J., Urgen M., Celis J.-P. (2014). Mechanical and frictional behaviour of nano-porous anodised aluminium. Mater. Chem. Phys..

[26] Guezmil M., Bensalah W., Khalladi A., Elleuch K., Depetris-Wery M., Ayedi H.F. (2015). Friction coefficient and microhardness of anodized aluminum alloys under different elaboration conditions. Trans. Nonferrous Met. Soc. China.

[27] Vengatesh P., Kulandainathan M.A. (2015). Hierarchically ordered self-lubricating superhydrophobic anodized aluminum surfaces with enhanced corrosion resistance. ACS Appl. Mater. Interfaces.

[28] Dejun K., Jinchun W., Hao L. (2016). Friction and wear performances of 7475 aluminium alloy after anodic oxidation. Rare Met. Mater. Eng..

[29] Lu J., Wei G., Yu Y., Guo C., Jiang L. (2018). Aluminum alloy AA2024 anodized from the mixed acid system with enhanced mechanical properties. Surf. Interfaces.

[30] Chen L., Liu Z., Wang B., Song Q., Wan Y., Chen L. (2019). Surface characterization and tribological performance of anodizing micro-textured aluminum-silicon alloys. Materials (Basel).

[31] Benea L., Dumitrascu V. (2019). Enhancement in sustained friction and wear resistance of nanoporous aluminum oxide films obtained by controlled electrochemical oxidation process. RSC Adv..

[32] Wernick S., Pinner R., Mason R.B. (1957). The surface treatment and finishing of aluminium and its alloys. J. Electrochem. Soc..

[33] Hu N., Ge S., Fang L. (2011). Tribological properties of nano-porous anodic aluminum oxide template. J. Cent. South Univ. Technol..

[34] Lee G.-S., hoon Choi J., Choi Y.C., Bu S.D., Lee Y.-Z. (2011). Tribological effects of pores on an anodized Al alloy surface as lubricant reservoir. Curr. Appl. Phys..

[35] Xu T., Chen J., Zhao J., Dang H. (1996). The pore-enlargement and self-lubrication treatment of anodic oxide film of aluminum. Wear.

[36] Takaya M., Hashimoto K., Toda Y., Maejima M. (2003). Novel tribological properties of anodic oxide coating of aluminum impregnated with iodine compound. Surf. Coat. Technol..

[37] Tu J.P., Jiang C.X., Guo S.Y., Zhao X.B., Fu M.F. (2005). Tribological properties of aligned film of amorphous carbon nanorods on AAO membrane in different environments. Wear.

[38] Skeldon P., Wang H., Thompson G. (1997). Formation and characterization of self-lubricating MoS_2_ precursor films on anodized aluminium. Wear.

[39] Maejima M., Saruwatari K., Takaya M. (2000). Friction behaviour of anodic oxide film on aluminum impregnated with molybdenum sulfide compounds. Surf. Coat. Technol..

[40] Liew K.W., Chia S.Y., Kok C.K., Low K.O. (2013). Evaluation on tribological design coatings of Al_2_O_3_, Ni–P–PTFE and MoS_2_ on aluminium alloy 7075 under oil lubrication. Mater. Des..

[41] Jiang C.X., Tu J.P., Guo S.Y., Fu M.F., Zhao X.B. (2005). Friction properties of oil-infiltrated porous AAO film on an aluminum substrate. Acta Metall. Sin. (Engl. Lett.).

[42] Mitani M. (2008). Method for Surface Treatment of Aluminum or Aluminum Alloy. European Patent.

[43] Santecchia E., Cabibbo M., Hamouda A.M.S., Musharavati F., Popelka A., Spigarelli S. (2020). Dry sliding tribological properties of a hard anodized AA6082 aluminum alloy. Metals (Basel).

[44] Howard S.M., Ellingham Diagrams (2006). Standard Gibb’s Energies of Formation for Chlorides, Nitrades, Oxides and other Compounds. Free Energies of Formation of Binary Compounds.

[45] Zhu B., Seifeddine S., Persson P.O.Å., Jarfors A.E.W., Leisner P., Zanella C. (2016). A study of formation and growth of the anodised surface layer on cast Al-Si alloys based on different analytical techniques. Mater. Des..

[46] Fratila-Apachitei L., Tichelaar F.D., Thompson G.E., Terryn H., Skeldon P., Duszczyk J., Katgerman L. (2004). A transmission electron microscopy study of hard anodic oxide layers on AlSi(Cu) alloys. Electrochim. Acta.

[47] Alemi A., Hosseinpour Z., Dolatyari M., Bakhtiari A. (2012). Boehmite (γ-AlOOH) nanoparticles: Hydrothermal synthesis, characterization, pH-controlled morphologies, optical properties, and DFT calculations. Phys. Status Solidi.

[48] Kloprogge J.T., Ruan H.D., Frost R.L. (2002). Thermal decomposition of bauxite minerals: Infrared emission spectroscopy of gibbsite, boehmite and diaspore. J. Mater. Sci..

[49] Russell J.D., Farmer V.C., Lewis D.G. (1978). Lattice vibrations of boehmite (γ-AlOOH): Evidence for a C122v rather than a D172h space group. Spectrochim. Acta Part A Mol. Spectrosc..

[50] Kiss A.B., Keresztury G., Farkas L. (1980). Raman and i.r. spectra and structure of boehmite (γ-AlOOH). evidence for the recently discarded D172h space group. Spectrochim. Acta Part A Mol. Spectrosc..

[51] Farmer C. (1974). The Infrared Spectra of Minerals.

[52] Van Der Marel R., Van Der Marel H.W., Beutelspacher H. (1974). Atlas of Infrared Spectroscopy of Clay Minerals and Thesir Admixtures.

[53] Diggle J.W., Downie T.C., Goulding C.W. (1969). Anodic oxide films on aluminum. Chem. Rev..

[54] Ofoegbu S.U., Fernandes F.A.O., Pereira A.B. (2020). The sealing step in aluminum anodizing: A focus on sustainable strategies for enhancing both energy efficiency and corrosion resistance. Coatings.

[55] Bull S.J., Berasetegui E.G. (2006). An overview of the potential of quantitative coating adhesion measurement by scratch testing. Tribol. Int..

[56] Barletta M., Rubino G., Tagliaferri V., Trovalusci F., Vesco S. (2014). Wood-reinforced polyphthalamide resins: Multifunctional composite coating for metal substrates. Int. J. Polym. Sci..

[57] Bull S.J. (1997). Failure mode maps in the thin film scratch adhesion test. Tribol. Int..

[58] Burnett P.J., Rickerby D.S. (1987). The relationship between hardness and scratch adhesion. Thin Solid Films.

[59] Sola R., Tonelli L., Shashkov P., Bogdanoff T.H., Martini C. (2020). Anodizing of AA6082-T5 by conventional and innovative treatments: Microstructural characterization and dry sliding behaviour. Wear.

[60] Yerokhin A.L., Nie X., Leyland A., Matthews A., Dowey S.J. (1999). Plasma electrolysis for surface engineering. Surf. Coat. Technol..

[61] Zhu S., Cheng J., Qiao Z., Yang J. (2019). High temperature solid-lubricating materials: A review. Tribol. Int..

[62] Lu J., Xue Q., Zhang G. (1998). Effect of silver on the sliding friction and wear behavior of CeF_3_ compact at elevated temperatures. Wear.

